# Corneal and Epithelial Thickness Mapping: Comparison of Enhanced Spectral-Domain- and Spectral-Domain-Optical Coherence Tomography

**DOI:** 10.1155/2021/3444083

**Published:** 2021-10-05

**Authors:** Cristina Georgeon, Ilanite Marciano, Roxane Cuyaubère, Otman Sandali, Nacim Bouheraoua, Vincent Borderie

**Affiliations:** GRC 32,Transplantation et Thérapies Innovantes de La Cornée, Sorbonne Université, Centre Hospitalier National D'Ophtalmologie des Quinze-Vingts, Paris, France

## Abstract

**Objective:**

To compare the results and repeatability of the corneal thickness (CT) and epithelial thickness (ET) maps provided by Enhanced Spectral-Domain-Optical Coherence Tomography with those of Spectral-Domain-OCT in normal eyes.

**Methods:**

30 normal eyes of 30 patients were assessed by 3 trained operators with ESD-OCT and SD-OCT.

**Results:**

The central and minimum ET obtained with both devices were correlated: central ET, *r* = 0.86, *p* < 0.05; minimum ET, *r* = 0.72, *p* < 0.05. Compared with SD-OCT, ESD-OCT tended to underestimate these figures by 1.4 and 1.9 *μ*m on average. The central and minimum CT obtained with both devices were strongly correlated: central CT, *r* = 0.994, *p* < 0.05; minimum CT, *r* = 0.995, *p* < 0.05. ESD-OCT tended to overestimate these figures by 11 and 14 *μ*m on average. Repeatability was good for both devices with a mean coefficient of variation of measurements <6% for ET and <2% for CT. Interoperator variability (standard deviation and COV) was significantly higher for ESD-OCT than for SD-OCT for all local epithelial thicknesses and significantly lower for the central CT and several local corneal thicknesses, whereas no significant differences between both technologies were found for the central and minimum ET and the minimum CT.

**Conclusion:**

ESD-OCT and SD-OCT provide reproducible measurements of CT and ET in normal corneas with a strong correlation between both technologies. However, both technologies are not interchangeable when the main thickness parameters (i.e., central and minimum CT and minimum ET) are used for diagnosing early keratoconus or calculating the expected residual stromal bed thickness before corneal refractive surgery or anterior lamellar keratoplasty.

## 1. Introduction

Corneal thickness and epithelial thickness mapping is a recent useful and key tool for diagnosing and monitoring corneal conditions. It was first developed using high-frequency ultrasound thickness maps that were shown to be relevant for the diagnosis of keratoconus [1, 2]. Further development of noncontact Spectral-Domain-Optical Coherence Tomography (SD-OCT) led to the widespread use of corneal thickness and epithelial thickness maps for the diagnosis of keratoconus and postoperative corneal ectasia including early diagnosis and classification, evaluation of keratoconus progression, and hydrops risk assessment, diagnosis of corneal epithelial basement membrane dystrophy, and assessment of corneal epithelial remodeling after cross-linking or refractive surgery [3–6].

Recently, the Enhanced Spectral-Domain-OCT (ESD-OCT) technology was developed for anterior segment imaging. This technology allows the whole anterior segment to be visualized in a single scan, and it has been coupled with corneal specular topography which permits a large number of data to be rapidly collected. Corneal thickness and epithelial thickness maps are also available with this technology.

The SD-OCT and ESD-OCT technologies have been assessed for reproducibility of measurements which is high for both [7–13]. We wondered whether the mapping information provided by ESD-OCT was as precise as the one provided by SD-OCT and whether both technologies were interchangeable. To address this issue, the present study aimed at comparing the results and repeatability of the corneal thickness and epithelial thickness maps provided by ESD-OCT with those of SD-OCT in normal eyes.

## 2. Materials and Methods

### 2.1. Patients

We prospectively analyzed 30 healthy eyes of 30 volunteers, including ametropia. Inclusion criteria were the following: spectacle-corrected visual acuity of 20/20 or higher, clear cornea, normal intraocular pressure in the range of 10–20 mmHg, and absence of anterior and posterior segment anomalies. Patients with previous corneal surgery, previous or current corneal disease or other diseases of the eye, or contact lens wear were excluded from the study. The patients' mean age was 26 ± 8 years, at a range of 19–57. The Enhanced Spectral-Domain-Optical Coherence Topography examination was performed before any contact with the eye (i.e., application of eye drops). All individuals previously underwent vision tests, slit-lamp examination, and noncontact tonometry. Following Enhanced Spectral-Domain-OCT examination all individuals underwent Spectral-Domain-OCT examination.

### 2.2. Enhanced Spectral-Domain-Optical Coherence Tomography

We acquired 9 mm-wide ESD-OCT scans (MS39, CSO, Firenze, Italy). Scans had an axial resolution of 3.5 *μ*m, a transverse resolution of 35 *μ*m, and a maximal depth of 7.5 mm. The proper examination centration was achieved by focusing the OCT scan on the corneal vertex detected by a hyper-reflective wide line perpendicular to the corneal surface. This line is obtained only when the OCT signal is perpendicular to the corneal vertex.

### 2.3. Spectral-Domain-Optical Coherence Tomography

We acquired 6 mm-wide SD-OCT scans (Optovue RTVue-100®; Optovue Inc®, Fremont, California, USA) with the long corneal adaptor module (CAM-L) in the center of the cornea. Scans had an axial resolution of 5 *μ*m and a transverse resolution of 15 *μ*m. Corneal adaptor module software automatically processes the OCT scans to provide the epithelial thickness map in the central 6 mm. The proper examination centration was achieved by focusing the OCT scan on the corneal vertex detected by a hyper-reflective wide line perpendicular to the corneal surface.

For both OCT devices, corneal epithelial thickness is measured as the distance between the air-tear and the epithelium-Bowman's layer interfaces perpendicular to the anterior surface at the point of measurement. An epithelial thickness profile is generated from each meridional cross section.

### 2.4. Methods

Three successive examinations were performed by three trained operators (1 examination per operator). Exams with missing data in the 6 mm zone were not taken into consideration.

To assess reproducibility, we calculated the interoperator standard deviation (SD) and coefficient of variation (COV) of the 3 successive individual measurements.

Our research adhered to the tenets of the “Declaration Of Helsinki.” The study was approved by an ethics committee, and informed consent was obtained from volunteers.

### 2.5. Statistical Analysis

The statistical analysis was performed with Statistica (version 6.0, Oklahoma, USA). The threshold value for the significance was defined as *p* < 0.05. Correlation between the two OCT devices was assessed with the Pearson correlation coefficient. Differences between measures provided by both devices were assessed with the paired *t*-test.

## 3. Results

### 3.1. Central and Minimal Thicknesses

#### 3.1.1. Corneal Epithelial Thickness

**Figure 1:**
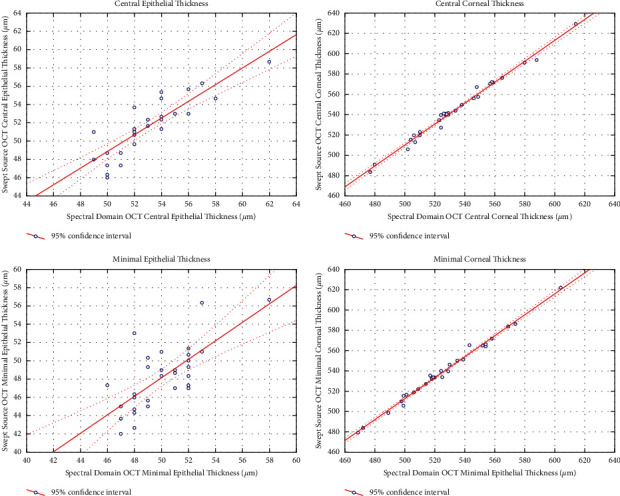
Correlation between Enhanced Spectral-Domain-OCT measurements and Spectral-Domain-OCT measurements of the epithelial and corneal thicknesses.

The central and minimal corneal epithelial thicknesses obtained with both OCT devices were significantly correlated: central corneal epithelial thickness, *r* = 0.86, *p* < 0.05; minimal corneal epithelial thickness, *r* = 0.72, *p* < 0.05 (Figure 1). Compared with the Spectral-Domain-OCT device, the Enhanced Spectral-Domain-OCT device provided significantly lower corneal epithelial thicknesses with a 1.4 *μ*m mean difference between both devices for the central corneal epithelial thickness (*p*=0.00003) and a 1.9 *μ*m mean difference for the minimal corneal epithelial thickness (*p*=0.0003). The power (i.e., the probability that a 1.5 *μ*m difference in corneal epithelial thickness between SD-OCT and ESD-OCT measurements would be significant) was 85% for central measurements and 55% for peripheral measurements.

#### 3.1.2. Corneal Thickness

The central and minimal corneal thicknesses obtained with both OCT devices were strongly correlated: central corneal thickness, *r* = 0.994, *p* < 0.05; minimal corneal thickness, *r* = 0.995, *p* < 0.05 (Figure 1). Compared with the Spectral-Domain-OCT device, the Enhanced Spectral-Domain-OCT device provided significantly higher corneal thicknesses (*p*=0.000001) with a 11 *μ*m mean difference between both devices for the central corneal thickness and a 14 *μ*m mean difference for the minimal corneal thickness. The power (i.e., the probability that a 5 *μ*m difference in corneal thickness between SD-OCT and ESD-OCT measurements would be significant) was 92% for central measurements and 75% for peripheral measurements.

### 3.2. Thickness Maps

#### 3.2.1. Corneal Epithelial Thickness

**Figure 2:**
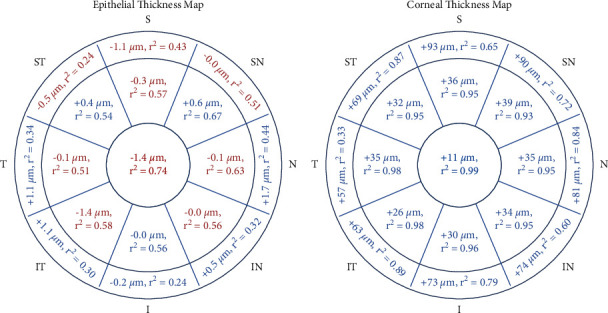
Differences and correlation between Enhanced Spectral-Domain-OCT measurements and Spectral-Domain-OCT measurements of the epithelial and corneal thicknesses in the 6 mm zone.

Significant differences in the 6 mm epithelial thicknesses were observed in all areas with either higher or lower values obtained with the ESD-OCT device compared with the SD-OCT device (Figure 2). Correlation between both device measurements was significant in all areas. However, a stronger correlation was observed in the 2 mm central area compared with the 2–5 mm peripheral areas and in the 2–5 mm peripheral areas compared with the 5–6 mm peripheral areas (Figure 2).

#### 3.2.2. Corneal Thickness

Compared with the Spectral-Domain-OCT device, the Enhanced Spectral-Domain-OCT device provided significantly higher corneal thicknesses in all areas. Lower differences between these figures were observed in the 2 mm central area compared with the 2–5 mm peripheral areas and in the 2–5 mm peripheral areas compared with the 5–6 mm peripheral areas (Figure 2). Correlation between both device measurements was significant in all areas. However, a stronger correlation was observed in the 2 mm central area compared with the 2–5 mm peripheral areas and in the 2–5 mm peripheral areas compared with the 5–6 mm peripheral areas (Figure 2). Conversely, no significant differences between both technologies were found for the central and minimal epithelial thicknesses and the minimum corneal thickness.

### 3.3. Reproducibility of Thickness Measures

Tables 1 and 2 show the interobserver reproducibility of thickness assessment. Measurements were performed by 3 experienced operators. Interoperator variability of measurements assessed with the mean standard deviation and mean coefficient of variation was low for both devices showing high reproducibility of thickness measurement achieved with both devices. However, variability was significantly higher for the Enhanced Spectral-Domain-OCT device than for the Spectral-Domain-OCT device for all local epithelial thicknesses and lower for the central corneal thickness and the local corneal thickness in various locations.

## 4. Discussion

The main purpose of this study was to compare two advanced OCT technologies providing corneal mapping data and to assess their reproducibility in normal corneas.

Regarding the two main parameters featuring corneal thickness (i.e., the central and minimum corneal thicknesses), we found a strong correlation between both technologies. However, the Enhanced Spectral-Domain technology tends to overestimate these figures by 11 and 14 *μ*m on average compared with the Spectral-Domain technology. The correlation between both technologies was weaker for the central and minimum corneal epithelial thicknesses. Compared with the Spectral-Domain technology, the Enhanced Spectral-Domain technology tends to underestimate these figures by 1.4 and 1.9 *μ*m. Consequently, both technologies are not interchangeable when these main thickness parameters are used for diagnosing early keratoconus or calculating the expected residual stromal bed thickness before corneal refractive surgery or anterior lamellar keratoplasty.

Reproducibility of thickness measurements was good for both devices with a mean coefficient of variation of <6% for the corneal epithelial thickness measures and <2% for the corneal thickness measures. The precision of corneal epithelial thickness measurements obtained with both OCT technologies appears to be close to that of Artemis very-high-frequency digital ultrasound [2]. However, the former technologies are truly noncontact with no need for a liquid interface between the cornea and the device as is the case for the Artemis device. The precision of the ET measurement we observed is also consistent with that measured with various OCT devices [3, 12, 13]. However, we did not observe a decreased precision in peripheral areas as was previously reported [3]. The Enhanced Spectral-Domain technology was associated with better reproducibility for the central corneal thickness, whereas no significant differences between both technologies were observed for the minimum corneal thickness and the main corneal epithelial thickness parameters. Reproducibility of the Enhanced Spectral-Domain technology in keratoconus eyes has been reported recently [11]. The mean coefficient of variation was 3.17% for the central epithelial thickness and 1.06% for the thinnest corneal thickness. These figures were, respectively, 1.7% and 0.3% in normal eyes in the present study.

Regarding the local peripheral thicknesses, the correlation between both technologies tended to decrease with the distance from the center of the cornea. One reason for this finding could be that the assessed areas outside the central zone could be different in both devices. The local peripheral measures provided by ESD-OCT and SD-OCT are not interchangeable. Interestingly the Enhanced Spectral-Domain technology provided better reproducibility of local peripheral corneal epithelial thickness measures compared with the Spectral-Domain technology. This finding might be relevant when the corneal epithelial map is used for diagnosing ocular surface disorders such as the corneal epithelial basement membrane dystrophy, limbal deficiency, or dry eye [9, 14, 15]. As the incidence angle between the OCT signal and the corneal surface increases with the distance from the center of the cornea, one can wonder whether this angle would influence the measurement and whether the Enhanced Spectral-Domain technology would be less dependent on this angle which could result in better reproducibility of local peripheral corneal epithelial thickness.

Advantages of the Enhanced Spectral-Domain technology combined with specular topography include analysis of a larger corneal area which allows peripheral doughnut patterns to be detected and the possibility to detect co-localized posterior surface ectasia and corneal/epithelial thinning. These advantages might be useful for keratoconus diagnosis.

The number of patients included in the present study (30) can be considered a study limitation. However, the a posteriori power calculation showed that the probability to detect a 1.5 *μ*m difference in epithelial thickness and a 5 *μ*m difference in corneal thickness was quite acceptable.

In conclusion, ESD-OCT and SD-OCT provide reproducible measurements of corneal thickness and corneal epithelial thickness in normal corneas with a strong correlation between both technologies. However, they are not interchangeable and diagnosis threshold values determined with one technology cannot be used with the other technology.

## Figures and Tables

**Table 1:** Interobserver reproducibility of corneal epithelial thickness assessment with Enhanced Spectral-Domain and Spectral-Domain-Optical Coherence Tomography in normal eyes.

	Mean standard deviation (*μ*m)			Mean coefficient of variation (%)		
	ESD-OCT	SD-OCT	*p*	ESD-OCT	SD-OCT	*p*
Central	0.88	0.73	0.34	1.7	1.3	0.24
Minimal	1.18	1.17	0.98	2.4	2.4	0.97
Maximal	1.06	1.032	0.89	1.8	1.8	0.96
2–5 mm superior	**1.79**	**0.87**	0.001	**3.4**	**1.6**	0.0006
2–5 mm supero-nasal	**1.59**	**0.76**	0.0004	**3.0**	**1.4**	0.0002
2–5 mm nasal	**1.46**	**0.82**	0.002	**2.7**	**1.5**	0.001
2–5 mm infero-nasal	**1.14**	**0.75**	0.02	**2.1**	**1.4**	0.01
2–5 mm inferior	**1.36**	**0.77**	0.002	**2.5**	**1.4**	0.001
2–5 mm infero-temporal	**1.36**	**0.69**	0.002	**2.5**	**1.3**	0.001
2–5 mm temporal	**1.44**	**0.70**	0.0001	**2.8**	**1.3**	0.00005
2–5 mm supero-temporal	**1.67**	**0.93**	0.005	**3.2**	**1.8**	0.003
5–6 mm superior	**2.82**	**1.06**	0.002	**5.7**	**2.1**	0.002
5–6 mm supero-nasal	**2.16**	**0.93**	0.000007	**4.1**	**1.8**	0.000004
5–6 mm nasal	**1.43**	**0.87**	0.001	**2.6**	**1.6**	0.002
5–6 mm infero-nasal	**1.66**	**0.86**	0.003	**3.0**	**1.6**	0.004
5–6 mm inferior	**1.46**	**0.75**	0.002	**2.6**	**1.4**	0.002
5–6 mm infero-temporal	**1.56**	**0.83**	0.003	**2.8**	**1.6**	0.006
5–6 mm temporal	**1.88**	**0.91**	0.0006	**3.6**	**1.7**	0.0009
5–6 mm supero-temporal	**1.79**	**1.22**	0.045	**3.5**	**2.4**	0.045

Each measurement was performed by 3 trained operators in 30 eyes of 30 patients. Data in bold indicate significant differences between ESD-OCT and SD-OCT.

**Table 2:** Interobserver reproducibility of corneal thickness assessment with Enhanced Spectral-Domain and Spectral-Domain-Optical Coherence Tomography in normal eyes.

	Mean standard deviation (*μ*m)			Mean coefficient of variation (%)		
	ESD-OCT	SD-OCT	*p*	ESD-OCT	SD-OCT	*p*
Central	**1.21**	**1.99**	0.03	**0.2**	**0.4**	0.03
Minimal	1.77	1.96	0.77	0.3	0.4	0.73
2–5 mm superior	6.24	4.94	0.63	1.1	0.8	0.66
2–5 mm supero-nasal	**2.24**	**4.58**	0.001	**0.4**	**0.8**	0.0005
2–5 mm nasal	**2.43**	**3.37**	0.036	**0.4**	**0.6**	0.01
2–5 mm infero-nasal	2.11	2.66	0.19	0.4	0.5	0.11
2–5 mm inferior	2.28	3.28	0.14	0.4	0.6	0.11
2–5 mm infero-temporal	2.32	2.76	0.44	0.4	0.5	0.40
2–5 mm temporal	**2.13**	**3.54**	0.004	**0.4**	**0.6**	0.003
2–5 mm supero-temporal	**2.69**	**4.88**	0.004	**0.5**	**0.9**	0.002
5–6 mm superior	7.50	7.03	0.88	1.1	1.1	0.88
5–6 mm supero-nasal	5.11	7.97	0.22	0.8	1.3	0.11
5–6 mm nasal	3.64	6.18	0.09	**0.5**	**1.0**	0.046
5–6 mm infero-nasal	9.66	5.66	0.45	1.5	1.0	0.53
5–6 mm inferior	**2.52**	**7.44**	0.008	**0.4**	**1.3**	0.006
5–6 mm infero-temporal	3.36	6.37	0.08	0.5	1.1	0.06
5–6 mm temporal	3.71	4.67	0.32	0.6	0.8	0.19
5–6 mm supero-temporal	7.27	6.72	0.78	1.1	1.1	0.93

Each measurement was performed by 3 trained operators in 30 eyes of 30 patients. Data in bold indicate significant differences between ESD-OCT and SD-OCT.

## Data Availability

Data are available upon request to the corresponding author.

## Abbreviations

SD-OCT: Spectral-Domain-Optical Coherence Tomography
ESD-OCT: Enhanced Spectral-Domain-Optical Coherence Tomography.
